# The combination effects of body acupuncture and auricular acupressure compared to sham acupuncture for body weight control: study protocol for a randomized controlled trial

**DOI:** 10.1186/s13063-016-1458-2

**Published:** 2016-07-25

**Authors:** Linda L. D. Zhong, Wai Kun, Tsz Fung Lam, Shi Ping Zhang, Jun Jun Yang, Tat Chi Ziea, Bacon Ng, Zhao Xiang Bian

**Affiliations:** 1 Hong Kong Chinese Medicine Study Centre, Hong Kong Baptist University, AAB 105, Hong Kong Baptist University, Kowloon Tong, Kowloon Hong Kong; 2 School of Chinese Medicine, Hong Kong Baptist University, 3/F, Jockey Club School of Chinese Medicine Building, 7 Baptist University Road, Kowloon Tong, Kowloon Hong Kong; 3 Chinese Medicine Department, Hong Kong Hospital Authority, Kowloon, Hong Kong

## Abstract

**Background:**

Obesity is an increasingly prevalent chronic condition that is associated with serious morbidity and mortality. Excess body weight is a risk factor contributing to diseases such as hypertension, heart disease, hypercholesterolaemia, diabetes mellitus, cerebrovascular disease, gall bladder disease, and some types of cancer. Almost all the Western anti-obesity drugs have adverse effects or body weight is regained upon cessation of therapy. Recent studies have found that acupuncture had a similar efficacy as the Western anti-obesity drugs with fewer reported adverse effects. However, these conclusions were limited due to the small sample size and low quality of methodologies of these studies. Therefore, we design this study to explore the effectiveness and safety of acupuncture on weight control.

**Methods/design:**

This is a pilot single-blinded, randomized, sham-controlled trial on acupuncture for body weight control. Seventy-two participants are randomly assigned to the acupuncture group or the control group. Tianshu (ST-25), Daheng (SP-15), Daimai (GB-26), Qihai (CV-6), Zhongwan (CV-12), Zusanli (ST-36), Fenglong (ST-40), and Sanyinjiao (SP-6) are selected as acupuncture points. For the acupuncture group, disposable acupuncture needles will be inserted at a depth of 10–25 mm into the points and electrical stimulation with dense-disperse waves at 50 Hz and 10 V will be applied on the abdominal points. The bodily needles will be retained for 30 minutes. For subjects assigned to the control group, Streitberger’s non-invasive acupuncture needles will be applied to serve as the sham control at the same acupoints with the same stimulation modality, except that the needles are only adhered to the skin instead of inserted. The duration of the treatment is 8 weeks with two sessions per week, and the follow-up period is 8 weeks. The primary outcome is the change in body weight before and after treatment. The secondary outcomes include changes in body mass index, waist circumference, hip circumference, and body fat percentage during the treatment and follow-up period.

**Discussion:**

The study will compare the efficacy and safety of acupuncture with sham acupuncture on weight control, in the hope of obtaining evidence for utilizing acupuncture for body weight control.

**Trial registration:**

NCT02516878. Registered on 30 July 2015.

## Background

Obesity is an increasingly prevalent chronic condition that is associated with serious morbidity and mortality [1]. According to the statistical data of the Centre for Health Protection, Hong Kong SAR in 2013, 36.6 % of the population aged 18–64 were classified as overweight or obese (body mass index, BMI ≥ 23), including 18.8 % as obese (BMI ≥ 25) among all the population. Excess body weight is the sixth most important risk factor contributing to the overall burden of disease worldwide, such as hypertension, heart disease, hypercholesterolaemia, diabetes mellitus, cerebrovascular disease, gall bladder disease, and some types of cancer [2, 3]. Obesity is becoming a global epidemic and common health problem. The weight control treatments commonly used nowadays include behavioural intervention, dietary intervention and physical activity, which require a higher level of self-discipline and take up to 6 months or longer to achieve significant weight change [4]; surgery like the duodenal–jejunal bypass liner, which may cause surgical complications [5]; and anti-obesity medications.

Currently, most of the Western anti-obesity drugs have adverse effects like oily discharge followed by flatus and faecal incontinence for orlistat [6]; headache, nausea and dizziness for lorcaserin [7] or body weight regaining upon cessation of therapy [8]. More people would like to seek help from Chinese herbal medicine (CHM) and/or acupuncture for body weight control, especially from acupuncture because it is safe and does not involve the intake of drugs [9].

Systematic reviews and randomized controlled trials have shown the beneficial effects of acupuncture, including body acupuncture and auricular acupressure [9, 10]. Acupuncture compared with placebo or lifestyle modification had more effectiveness in lowering body weight, BMI and waist circumference and it had a similar efficacy as the Western anti-obesity drugs with fewer reported adverse effects of insomnia, headache or gastrointestinal reactions [10, 11]. Auricular acupressure has been summarized as having the effect of appetite suppression on overweight patients [12], and its efficacy on weight control is higher when combined with body acupuncture [13].

However, these conclusions are limited due to the small sample size and low quality of the methodologies of these studies. Also, there is no restricted research to investigate the combination effects of body acupuncture and auricular acupressure compared to sham acupuncture for body weight control among Hong Kong’s Chinese population. Therefore, we design this single-blinded, randomized controlled clinical trial to explore the effectiveness, efficacy and safety of body acupuncture and auricular acupressure on weight control in Hong Kong.

### Objective

The aim of the study is to assess the efficacy and safety of body acupuncture and auricular acupressure compared to sham acupuncture on body weight control through a pilot randomized controlled study.

## Methods/design

### Study design

**Fig. 1:**
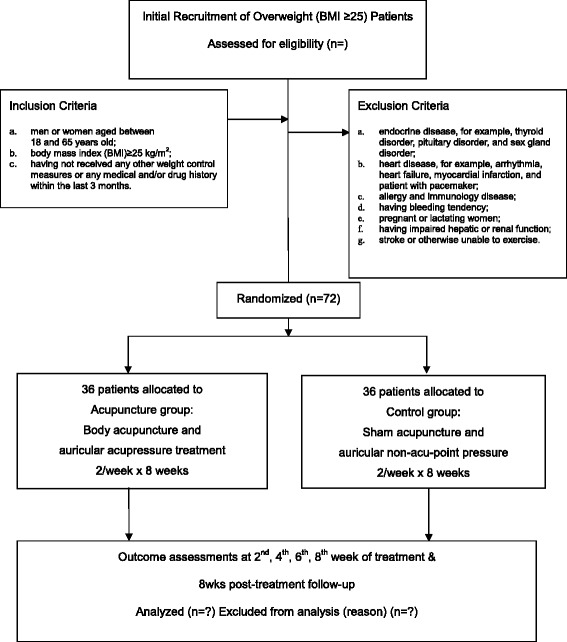
Participant flow diagram

This is a pilot single-blinded, randomized, sham-controlled trial on acupuncture for body weight control conducted at Hong Kong Baptist University Chinese Medicine Clinics. Seventy-two participants will be enrolled from the public through advertisements via the university’s website and newspaper. Subjects will be randomly assigned to the acupuncture group or the control group. Treatment will be given with two sessions per week for 8 weeks, and the post-treatment follow-up period will be 8 weeks. The primary outcome is the change in body weight before and after treatment. The total study period will be 16 weeks. The study protocol has been approved by the Hong Kong Baptist University Ethics Committee on the Use of Human Subjects for Teaching and Research (Approval no. HASC/13-14/0266) and registered in ClinicalTrials.gov (NCT02516878). The flow chart of the trial is shown in Fig. 1. The checklist for items in STRICTA 2010 is given in Table 1. Informed consent is obtained from each participant.

**Table 1:** Checklist for items in STRICTA 2010*

Item	Detail
1. Acupuncture rationale	1a) Style of acupuncture:
	According to systematic reviews and clinical experiences of our principal investigator and co-investigators. Manual and electro-acupuncture based on traditional Chinese medicine theory
	1b) Reasoning for treatment provided, based on historical context, literature sources, and/or consensus methods, with references where appropriate
	1c) Extent to which treatment was varied:
	Standard treatment is used. No variation of treatment among patients
2. Details of needling	2a) Number of needle insertions per subject per session (mean and range where relevant):
	14 needles
	2b) Names (or location if no standard name) of points used (uni/bilateral)
	Bilateral:
	Tianshu (ST-25), Daheng (SP-15), Daimai (GB-26),
	Zusanli (ST-36), Fenglong (ST-40), Sanyinjiao (SP-6)
	Unilateral:
	Qihai (CV-6), Zhongwan (CV-12)
	2c) Depth of insertion, based on a specified unit of measurement or on a particular tissue level:
	10–25 mm
	2d) Response sought (e.g. *de qi* or muscle twitch response):
	De qi
	2e) Needle stimulation (e.g. manual, electrical):
	Manual and electrical — dense-disperse waves at 50 Hz and 10 V
	2f) Needle retention time:
	30 min
	2g) Needle type (diameter, length and manufacturer or material):
	Disposable acupuncture needles (verum acupuncture needles asia-med Special No. 16 with 0.30 × 0.30 mm matching the Streitberger sham needles)
3. Treatment regimen	3a) Number of treatment sessions: 16 sessions
	3b) Frequency and duration of treatment sessions:
	2/week for 8 consecutive weeks
4. Other components of treatment	4a) Details of other interventions administered to the acupuncture group (e.g. moxibustion, cupping, herbs, exercises, lifestyle advice):
	Auricular acupressure with Semen Vaccariae embedded adhesive tape.
	Unilateral auricular points: Hunger, Shen men, Spleen and Stomach
	4b) Setting and context of treatment, including instructions to practitioners, and information and explanations to patients:
	University Clinics
	Participants will be informed about acupuncture treatment in the study as follows: “In this study, acupoints for weight control will be used based on related reports and clinical experience of our investigators.”
5. Practitioner background	5) Description of participating acupuncturists (qualification or professional affiliation, years in acupuncture practice, other relevant experience):
	Hong Kong registered Chinese medicine practitioners having at least 3 years of clinical experience, who have undergone training and are able to provide identical acupuncture treatment in accordance with a predefined protocol
6. Control or comparator interventions	6a) Rationale for the control or comparator in the context of the research question, with sources that justify this choice:
	To assess the efficacy and safety of body acupuncture and auricular acupuncture compared to sham acupuncture
	6b) Precise description of the control or comparator. If sham acupuncture or any other type of acupuncture-like control is used, provide details as for items 1 to 3 above
	- Style of acupuncture:
	Sham acupuncture + Semen Vaccariae embedded tape pressure on non-acupoints at auricular helix
	Table 1 Click here to download Table Table 1.docx
	- Number of needle insertions per subject per session:
	14 sham needles at the same acupoints as the treatment group, and 4 auricular helix points with embedded tape pressure
	- Depth of insertion:
	Needles are only adhered to the skin.
	- Needle retention time:
	30 min
	- Needle type
	Streitberger’s non-invasive acupuncture needles (Gauge 8 × 1.2”/0.30 × 30 mm)
	- Number of treatment sessions:
	16 sessions
	- Frequency and duration of treatment sessions:
	2/week for 8 consecutive weeks

*This checklist, which should be read in conjunction with the explanations of the STRICTA items, is designed to replace CONSORT 2010’s item 5 when reporting an acupuncture trial

### Participants

#### Setting

The study is conducted in the research and clinical centres, School of Chinese Medicine, Hong Kong Baptist University.

#### Inclusion criteria

Patients who meet all of the following criteria will be eligible for the study: men or women aged between 18 and 65 years old; with body mass index (BMI) ≥ 25 kg/m^2^; who have not received any other weight control measures or any medical and/or drug history within the last 3 months.

#### Exclusion criteria

Patients who meet any of the following criteria will be excluded from the study: those with endocrine disease, for example, thyroid disorder, pituitary disorder or sex gland disorder; those with heart disease, for example, arrhythmia, heart failure, myocardial infarction or patients with pacemakers; those with allergy and immunology disease; those having a bleeding tendency; pregnant or lactating women; those having impaired hepatic or renal function; those who have had a stroke or are otherwise unable to exercise.

### Interventions

#### Body acupuncture treatment

Acupuncture intervention will be conducted for two sessions per week over 8 consecutive weeks. According to a systematic review, the highly frequently used acupuncture points in body weight control trials are Zusanli (ST-36), Sanyinjiao (SP-6), Tianshu (ST-25), Fenglong (ST-40), Zhongwan (CV-12) and Qihai (CV-6) [9]. Among them, the traditional effects of the abdominal points are harmonizing gastrointestinal function, regulating Qi circulation and treating localized problems, which would be excessive adipose tissue deposition for the studied subjects. The traditional effects of the points at the lower limbs are stabilizing gastrointestinal function and enhancing fluid drainage. With the clinical experience of our principal investigator and co-investigators, eight body points are chosen: Tianshu (ST-25), Daheng (SP-15), Daimai (GB-26), Qihai (CV-6), Zhongwan (CV-12), Zusanli (ST-36), Fenglong (ST-40) and Sanyinjiao (SP-6). The details of acupoints and their functions are listed in Table 2. The acupuncture treatment will be conducted by a registered Chinese medicine practitioner with more than 6 years of Chinese medicine college education and at least 5 years of clinical experience.

**Table 2:** Acupoints of the body and their functions

Acupoint	Classical effects of stimulation
Tianshu (ST-25)	Restoring and harmonizing the flow of energy in the intestines; regulating the Qi; breaking up blocks
Daheng (SP-15)	Expelling cold in digestive system; regulating gastrointestinal functions
Daimai (GB-26)	Directing the Qi down into the lower body; stabilizing and harmonizing the lower tri-energizer (lower Jiao), thus draining dampness
Qihai (CV-6)	Supplementing and regulating Qi and Yang; stabilizing and nourishing the kidneys
Zhongwan (CV-12)	Regulating the stomach meridian, harmonizing stomach Qi; draining fluids
Zusanli (ST-36)	Stabilizing and regulating the stomach meridian, harmonizing Qi and the blood
Fenglong (ST-40)	Transforming body fluids; expelling phlegm
Sanyinjiao (SP-6)	Regulating the spleen, liver and kidney meridian; intensifying and dynamizing fluids circulation

Disposable acupuncture needles (verum acupuncture needles asia-med Special No. 16 0.30 × 30 mm matching the Streitberger sham needles) will be inserted at a depth of 10–25 mm into the points. As electro-acupuncture has been reviewed as having a higher efficiency in weight loss treatment [14], we will deliver electrical stimulation with dense-disperse waves at 50 Hz and 10 V through the electrical acupuncture stimulation instrument (ES-160 6-Channel Programmable Electro-acupuncture) to the abdominal points. The bodily needles will be retained for 30 minutes.

#### Auricular acupressure

**Fig. 2:**
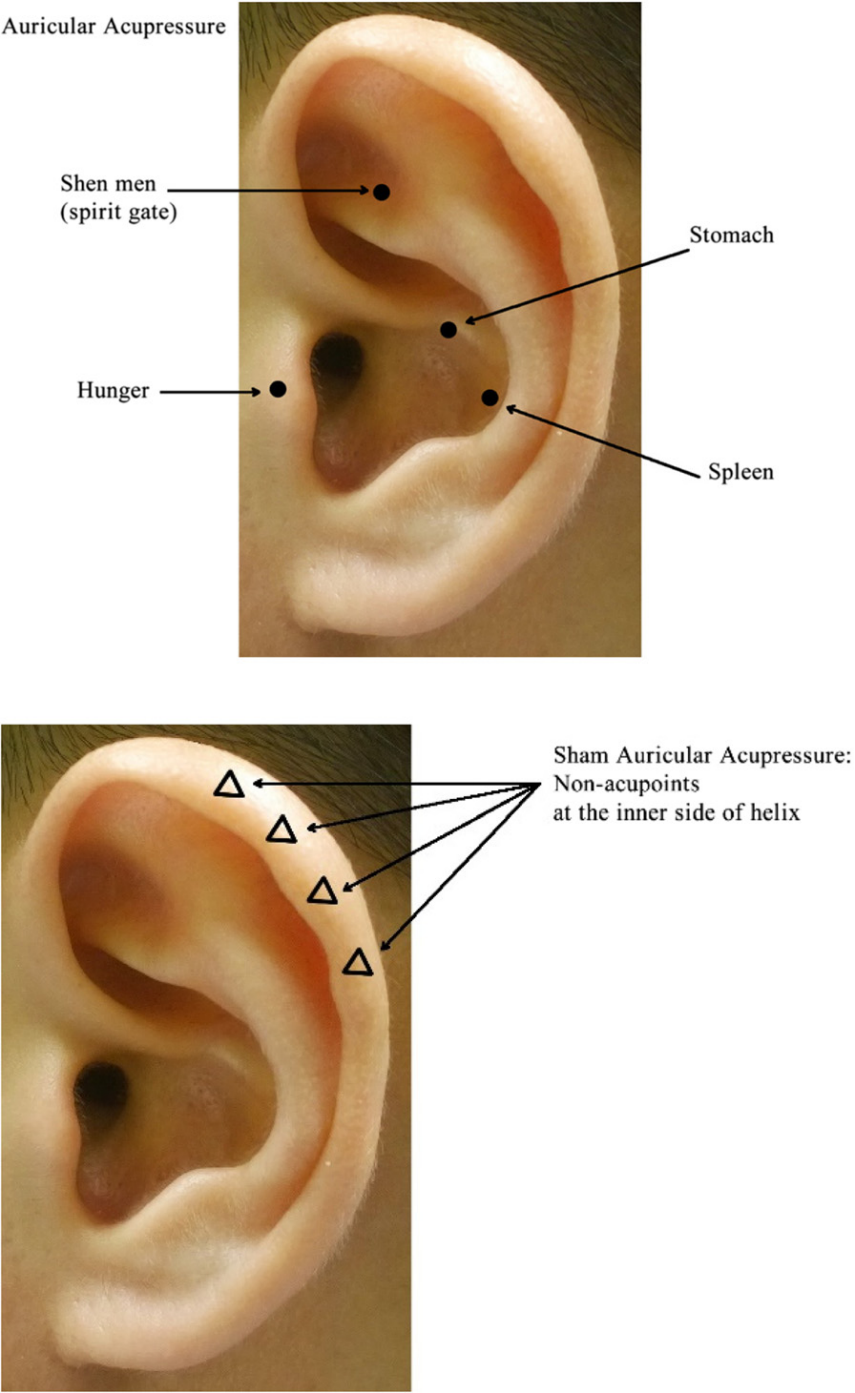
Auricular acupressure points and sham acupressure points

Participants of the treatment group will additionally receive unilateral auricular acupressure at four auricular points as Hunger, Shen men, Spleen and Stomach with Semen Vaccariae (Wang Bu Liu Xing) embedded within adhesive tape at each treatment session (Fig. 2). Acupressure will be self-applied by the subjects with repeated pressing of the tape with fingertips for 1 minute per point, thrice per day. The embedded tape will be retained in situ for 24 hours, and then the alternate ear will be treated at the next visit. The four points were most frequently used on overweight treatment as summarized by a systemic review [9]. Clinically, the ear points Spleen and Stomach are used for regulating gastrointestinal function; Shen men, meaning Spirit gate, is used for calming mental conditions; Hunger is used for suppressing the desire to eat and is reported to be particularly effective on weight loss [15].

#### Sham acupuncture

For subjects assigned to the control group, Streitberger’s non-invasive acupuncture needles (Gauge 8 × 1.2”/0.30 × 30 mm) will be applied to serve as a sham control at the same acupoints with the same stimulation modality, except that the needles are only adhered to the skin instead of being inserted [16]. The validity and credibility of this system has been well demonstrated [17, 18].

#### Sham auricular acupressure

The Semen Vaccariae embedded tape used in the treatment group will be applied on four non-acupoints at the helix unilaterally and retained for 24 hours, and then the alternate ear will be used at the next visit (Fig. 2). The helix region was suggested to be used as a control in auricular needling and was applied in previous clinical trials [19, 20].

All subjects will be advised to have a regular number of meals daily and not to have any snacks. Meals comprise one bowl of rice (210 g) for subjects > 70 kg and two-thirds of a bowl of rice (140 g) for those < 70 kg, with instructions to eat side dishes balanced with the rice. Also, subjects will be instructed not to perform any exercise other than that required for their daily work.

### Outcome measures

The primary outcome is the change in body weight before and after treatment. The secondary outcomes include changes in BMI, waist circumference, hip circumference and body fat percentage during the treatment and follow-up period. Besides at baseline (0 week), the numbers will be measured every 2 weeks. Both the body weight and body fat percentage will be measured with the Omron Karada Scan HBF-701.

Adverse events will be noted throughout the study, based on participant reports and laboratory tests (whole blood counts, renal and liver functions) if needed. All clinical adverse events will be recorded according to terms of intensity (mild, moderate or severe), duration, outcome and relationship to the study.

### Randomization assignment

Subjects of both groups will be randomly assigned to receive acupuncture (body and auricular acupressure) or control (sham) treatment. For randomization, simple, complete non-sequential random numbers will be generated in advance by a computer program in a block of four, and kept by the principal investigator (PI, ZXB). After a patient’s eligibility is confirmed, a randomization number which corresponds to the group allocation will be provided to the acupuncturist by the PI. This arrangement will ensure that the clinical assessor and participants are blinded to the allocation.

### Sample size

The sample size was calculated based on the primary endpoint of changes in body weight. A systematic review on acupuncture for obesity has shown that acupuncture significantly reduced the body weight with average weight loss (MD = 1.56 kg, 95 % CI = 0.74–2.38) [6]. Therefore, a sample size of 60 should be provided to achieve a significance level of α = 0.05 with a power (1 – β) of 90 % using a two-sample *t* test. The number of subjects increases to 72 when estimated 20 % dropouts are considered.

### Data processing and analysis

All efficacy and safety analyses will be conducted according to the intention-to-treat (ITT) principle. Missing values will be imputed by the last-observation-carried-forward method. The statistical analysis will be performed using the Statistical Package for the Social Sciences (SPSS) for Windows version 21.0. The statistical significance is defined as two-sided *P* value of < 0.05. Baseline characteristics will be reported as mean (SD). Baseline differences between the groups will be assessed with the use of Student’s *t* test for normally distributed continuous variables and the non-parametric Mann-Whitney *U* test for non-normally distributed variables. For categorical variables, the chi-squared test or Fisher’s exact test will be used. Comparisons between groups will be conducted by using an analysis of covariance (ANCOVA) with baseline as covariate. All items and subscales will be compared between groups every 4 weeks using ANCOVA, with treatment group as a factor in the model and baseline as the covariate. The changes from baseline to endpoint of treatment in scores will be tested with a repeated measures analysis of variance (ANOVA). Within-group differences will be assessed with a paired *t* test for normally distributed data and a Wilcoxon signed-rank test for non-normally distributed data.

## Discussion

This single-blinded, randomized controlled clinical trial aims to evaluate the efficacy and safety of acupuncture on body weight control in Hong Kong. It will be the first such study on the Hong Kong population and will obtain evidence for utilizing acupuncture in obesity treatment especially for patients with Hong Kong diet patterns and health conditions. In order to achieve a higher therapeutic efficacy, we will integrate electro-acupuncture with auricular acupressure. Except for acupuncture point stimulation on the body and ears, we will not apply any interventions such as CHM, diet or physical activities to the subjects. Therefore, the effects of acupuncture alone will be observed. The outcome measures will include the change in body weight and body fat percentage, which will provide data to us on analysis of the treatment’s potential benefit on body fat mass.

So far there is no study of this kind in Hong Kong. This pilot study will provide evidence for large-scaled research such as combined therapy on overweight subjects with CHM, diet control, physical training or behavioural intervention. Further research on the changes in hormones, the nervous system or psychological conditions can also be developed to specifically target the Hong Kong population, as there have only been overseas studies up to the present.

In this clinical trial, the selection of body and auricular points is standardized and utilized for every subject. This may be helpful for easier utilization of the treatment over different individuals, but the limitation is that the selection of acupuncture points is not based on syndrome differentiation, which is the main concern in traditional Chinese acupuncture. Another limitation is the small sample size, as the trial was proposed to be a pilot study for a later large-scaled clinical trial.

In conclusion, in this pilot study, a single-blinded, randomized controlled clinical trial will be conducted to evaluate the effectiveness, efficacy and safety of acupuncture on weight control in Hong Kong. This study will obtain the solid evidence for Chinese medicine practitioners (CMPs) to utilize acupuncture for obesity and will also provide a platform to offer research training opportunities for junior CMPs.

### Trial status

The participants are currently being recruited for the present study.

## Abbreviations

ANCOVA, analysis of covariance; ANOVA, analysis of variance; BMI, body mass index; CHM, Chinese herbal medicine; CMP, Chinese medicine practitioner; ITT, intention-to-treat
